# Cardiac Autoimmunity as a Novel Biomarker, Mediator, and Therapeutic Target of Heart Disease in Type 1 Diabetes

**DOI:** 10.1007/s11892-015-0598-1

**Published:** 2015-03-28

**Authors:** Myra A. Lipes, Alfonso Galderisi

**Affiliations:** 1 Joslin Diabetes Center, Harvard Medical School, 1 Joslin Place, Rm. 373, Boston, MA 02215 USA; 2 Department of Woman’s and Child’s Health, University of Padua, Via Giustiniani, 3, 35128 Padova, Italy

**Keywords:** Type 1 diabetes, Cardiovascular disease, Myocardial infarction, Myocarditis, Heart failure, Cardiomyopathy, Autoimmunity, Immune tolerance

## Abstract

Patients with type 1 diabetes (T1D) suffer excess mortality from cardiovascular disease (CVD) that has persisted despite substantial reductions in microvascular complications. Although T1D and type 2 diabetes (T2D) are etiologically distinct, it has generally been assumed that CVD in T1D is “the same disease” as that found in T2D. Here, we review the most recent epidemiological and clinical studies on heart disease in T1D, highlighting differences between CVD in T1D and T2D. In addition, we discuss experimental and clinical evidence for a post-myocardial infarction (MI) autoimmune heart syndrome in T1D, including the development of diagnostic assays which we believe can, for the first time, differentiate between heart disease in T1D and T2D. We postulate that a clinically unrecognized form of chronic myocardial inflammation (“myocarditis”) triggered by MI contributes to the poor CVD outcomes in T1D. These findings provide a conceptual shift in our understanding of CVD in T1D and have important diagnostic and therapeutic implications.

## Introduction

Cardiovascular disease (CVD) is the major cause of death in diabetes. Although CVD most commonly occurs in type 2 diabetes [1], which is far more prevalent than T1D, the risk of death from CVD is actually higher in T1D than in T2D [2]. This often under-appreciated excess CVD mortality in T1D is remarkable in occurring at a relatively young age, with the burden particularly striking for women [3, 4••]. Although the Diabetes Control and Complications Trial (DCCT)/Epidemiology of Diabetes Interventions and Complications (EDIC) Study clearly established poor glycemic control as a key mediator of CVD risk in T1D [5], hyperglycemia only explains a fraction of the overall risk [6]. While numerous factors related to diabetes have been implicated, none have been unique to T1D [7, 8]. In the absence of knowledge of disease-specific mechanisms, designing appropriate interventions and strategies for prevention of CVD in T1D has been problematic. Indeed, current management approaches of CVD in T1D have been largely extrapolated from experience in T2D [9••].

Although most published work on CVD has been focused on the role of inflammation in atherosclerosis [9••, 10, 11], this review will focus instead on the novel role of inflammation—specifically due to autoimmunity—as a potential mediator of cardiac dysfunction and heart failure in T1D [12••, 13••]. We will discuss our findings indicating that the same defects in the immune system that confer risk for T1D, also confer susceptibility to chronic myocarditis (chronic inflammation of heart muscle tissue) following a myocardial infarction (MI) and thereby may contribute to excessively poor CVD outcomes in T1D [12••, 14]. As a preliminary to these discussions, we will present an overview of recent epidemiological and clinical studies that have specifically focused on heart disease in T1D. We will then summarize differences between the pathogenesis of T1D and T2D with regard to CVD. We will then discuss how, starting with a serendipitous discovery in T1D-humanized mice [15], we generated a diverse body of findings that support an autoimmune “driver” for post-MI damage in T1D [12••, 13••, 14]. This review will focus on the key experiments and clinical evidence, including development of novel diagnostic methods which we believe can, for the first time, differentiate between heart disease in T1D and T2D. We believe these findings provide a conceptual shift of CVD in T1D, which is widely viewed as the “same disease” as CVD in T2D. Finally, we will discuss the implications of this new mechanistic framework on the development of targeted therapies to improve CVD outcomes in patients with T1D.

For the purposes of this review, CVD will be defined as coronary heart disease, the major form of CVD in T1D patients, and will not include cerebrovascular disease or peripheral vascular disease. We will not discuss the risk factors in T1D for atherosclerosis, the subject of a recent excellent review [9••].

## Cardiovascular Disease in Type 1 Diabetes: Scope of the Problem

It has long been recognized that patients with T1D suffer excess mortality from CVD, which accounts for 65–70 % of CVD deaths [8]. CVD is notable for occurring at a younger age in patients with T1D with the age-adjusted mortality rates between eight and 40 times that of the general population [8, 16–18] and far exceeding that observed in T2D [8, 16]. Indeed, a recent analysis of CVD risk in the large UK General Practice Research Database consisting of >7,400 patients with T1D with a mean age of 33 ± 14.5 years and a mean T1D duration of 15 ± 12 years showed that CVD events occurred on average 10–15 years earlier than age-matched non-diabetic control subjects [19]. CVD in T1D is also notable in that women have a paradoxically greater excess risk of death from CVD compared to men [3, 4••], with hazard ratios of death from CVD of 7.50 for women aged 35–49 years and 7.92 for women aged 50–64 years versus controls [4••].

Moreover, the results from the 30-year natural history studies of T1D in the Pittsburgh Epidemiology of Diabetes Complications Study highlight that despite a substantial decrease in other diabetic complications including end-stage renal disease, long considered the major driver of CVD in T1D [20], no concomitant reduction in morbidity and mortality from CVD has been reported, with CVD accounting for 40 % of deaths in T1D subjects after 20 years of disease [21]. This high mortality from CVD sharply contrasts with the extremely low mortality from CVD amongst young adults in the general population. These findings are confirmed, albeit to different extents, by several registry-based reports [3, 4••, 18, 19, 22–25].

Heart failure, one of the most severe complications of CVD, has recently been analyzed by Lind and colleagues in a nationwide Swedish observational study conducted on 20,985 relatively young T1D patients (mean age at baseline, 38.6 years). During 9 years of follow-up, they observed a 30 % higher risk of heart failure for each 1 % point increment in HbA1c, independently of MI [26••], the most frequent cause of heart failure in the general population [27]. This study further highlighted that men with T1D aged 41–45 years showed a similar incidence for heart failure (2.4 per 1000 person-years) to that in non-diabetic control subjects 15–20 years older (i.e., 2.1 per 1000 person-years in the 55–64 year age group) [26••]. This study was the first to show that heart failure is a major complication in relatively young patients with T1D.

More recently, this same Swedish group showed in another National Diabetes Registry-based study of relatively young patients with T1D (*n* = 33,915; mean age at baseline, 35.8 years; mean follow-up, 8.0 years) a stepwise increase in risk of death from CVD with poor glycemic control, up to a 10.5-fold greater in patients with mean HbA1C ≥9.7 % compared to the general population. An unexpected finding from this study was that even for T1D patients with good glycemic control (mean HbA1C ≤ 6.9 %) [4••], i.e., with HbA1C levels within the currently recommended on-target glycemic range [28] that was shown to confer long-term reduction in CVD events in the DCCT/EDIC study [5], the risk of death from CVD was still almost threefold greater than the risk in the general population. Since this T1D population was relatively young and did not generally have risk factors associated with T2D (e.g., obesity or hypertension), the mechanisms accounting for this excess risk of death from CVD could not be explained [4••].

Although hyperglycemia has been clearly established by the DCCT/EDIC [5] and other subsequent studies to be a key mediator of CVD risk in T1D [4••, 23, 25], hyperglycemia explained only a modest fraction of this overall risk and the mechanisms underlying excessive CVD mortality had not been defined [6]. Numerous factors related to the metabolic effects of diabetes have been implicated [8, 29]; however, none have been unique to T1D. This suggests that T1D bears disease-specific features that require deeper analysis.

## Type 1 and Type 2 Diabetes Are Etiologically Distinct

**Fig. 1:**
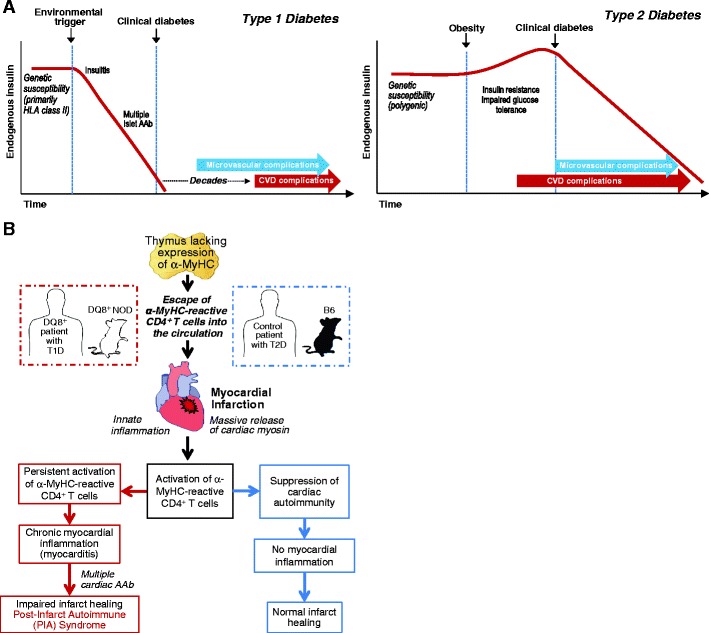
**a** Proposed pathogenesis of type 1 and type 2 diabetes as related to the differential timelines of their associated CVD complications. *HLA* human leucocyte antigen, *AAb* autoantibody. **b** Schematic of the pathogenesis of post-infarction autoimmunity (*PIA*) in T1D patients and humanized DQ8^+^NOD mice and how PIA is avoided in non-autoimmune-prone (“control”) T2D patients and non-autoimmune-prone B6 mice. *NOD* non-obese diabetic, *B6* C57BL/6, *α-MyHC* α-isoform of cardiac myosin heavy chain

Despite hyperglycemia representing a shared diagnostic phenotype between T1D and T2D, the two diseases are etiologically distinct. Type 2 diabetes, the most common form of diabetes, is typically diagnosed in midlife in patients who exhibit several risk factors for CVD including obesity, dyslipidemia, and hypertension [1]. This early metabolic imbalance is associated with increased peripheral insulin resistance on target tissues (adipose tissue, liver, and muscle) and increased fasting glucose levels that, however, remain below the diabetic limit for several years (Fig. 1a, right panel) [1, 28]. This asymptomatic phase (“prediabetes”) is associated with a systemic innate inflammatory process that involves vessels, particularly the coronary arteries. The adipose tissue, with its resident macrophages, and the liver are the main sources of proinflammatory cytokines and chemokines that reduce insulin sensitivity and promote the local inflammatory process implicated in the formation of the atherosclerotic plaque [30].

The systemic innate inflammation and the early involvement of vessels may explain why, in T2D, the first CVD event often occurs before the clinical diagnosis of diabetes (Fig. 1a), suggesting that hyperglycemia is just part of a complex systemic inflammatory process, characterized by peripheral insulin resistance with a relative insulin excess in the early phases of the disease and a wide production of inflammatory molecules by the adipocytes and liver (Fig. 1a) [1]. To this end, CVD in T2D has to be considered not only as a mere “complication” of hyperglycemia but a comorbidity that may occur independently of the progression to overt diabetic hyperglycemia [31].

In contrast, T1D is an autoimmune disease in which T cells of the adaptive immune system target and destroy pancreatic β cells, resulting in absolute insulin deficiency (Fig. 1a, left panel) [32, 33, 65]. Type 1 diabetes most commonly presents in childhood and adolescence, although it can occur at any age. Intriguingly, the incidence of T1D, especially in the very young (<5 years), has been increasing worldwide since the middle of the twentieth century and is predicted to double over the next decade [24, 34]. In contrast to patients with T2D, T1D patients typically experience their first MI after several decades of diabetes (Fig. 1) and there is strong evidence that the duration of T1D is highly predictive of CVD—with an increased risk of 34 % for every 10 years of disease and a substantially higher risk for individuals diagnosed with T1D in early childhood than for those as adults [26••]. Thus, the burden of CVD mortality in T1D will likely substantially increase in future years to come.

In humans and animal models, T1D is preceded by a state referred to as “insulitis” that is recognized pathologically as lymphocytic infiltration of pancreatic islets. Once insulitis is established, it can be detected indirectly by screening serum for antibodies to islet antigen components that include insulin, glutamic acid decarboxylase 65, tyrosine phosphatase IA-2, and more recently ZnT-8 [35]. These antibodies are present in 85–90 % of patients with newly diagnosed T1D, with the rate of β-cell destruction occurring more rapidly at younger ages [36]. The presence of multiple autoantibodies is particularly predictive of high risk for developing T1D [37, 38] and has been a useful surrogate marker of islet autoimmunity in diabetes prevention trials. In T2D, autoimmune destruction of β cells and expression of multiple islet autoantibodies do not occur.

A striking characteristic of T1D has been the almost exclusive development of the disease in individuals who bear human leukocyte antigen (HLA) class II alleles HLA-DQ2, HLA-DQ8, or both. These so-called high-risk alleles encode antigen-presenting molecules that present peptides to CD4 helper T cells of the adaptive immune response. The specific peptides presented by DQ2 and DQ8 are thought to explain the exquisite β-cell specificity of the autoimmune attack in T1D [32]. In addition, non-HLA genes that are associated with more global defects in immune regulation are also required for disease development and are thought to underlie the clustering of multiple autoimmune disorders in families and individuals with T1D [39]. These features are mirrored by non-obese diabetic (NOD) mice that spontaneously develop T1D and express the class II antigen-presenting molecule, I-A^g7^, which is closely related to human DQ8.

## HLA-DQ8-Humanized Mice Develop Premature Death Due to Autoimmune Myocarditis

A fortuitous observation in a humanized transgenic NOD mouse model a decade ago raised the possibility that autoimmune myocarditis might be included amongst the spectrum of organ-specific autoimmune comorbidities associated with T1D.

Two groups including ourselves independently reported that transgenic NOD mice expressing class II human leukocyte antigen (HLA) DQ8, in place of murine I-A^g7^, developed spontaneous autoimmune myocarditis [15, 40]. The disease process was characterized by lymphocytic infiltrates in the myocardium, high-titer circulating IgG autoantibodies against cardiac myosin heavy chain (hereafter, MyHC), and premature death due to congestive heart failure. Furthermore, our group found that the severity of myocarditis paralleled the incidence of T1D in three independently derived transgenic lines that differed only in their levels of DQ8 expression, suggesting that T1D and myocarditis, although clinically distinct, share common genetic pathways [15]. These findings raised the intriguing possibility that an unrecognized form of myocardial autoimmunity might contribute to the CVD burden in T1D.

Our group has since defined the cellular and molecular mechanisms of myocarditis in the DQ8^+^NOD model and then used this knowledge to develop sensitive and specific immunological assays for the detection of myocarditis in humans [12••, 13••]. These studies have shown evidence of an autoimmune heart disease process in T1D patients with CVD that are absent in T2D patients with CVD and will be discussed below.

## Myocarditis Is Mediated by Proinflammatory CD4 T Cells Reactive Against α-MyHC

In our analysis of the earliest immune responses in myocarditis in DQ8^+^NOD mice, we found that the first autoantibodies to appear were against the cardiac tissue-specific α-isoform of MyHC (α-MyHC), followed by reactivity to β-MyHC expressed in both cardiac and skeletal muscles, and then to other cardiac proteins [13••]. We proposed that this represented intra- and intermolecular epitope spreading and was an indicator of disease progression, in a manner analogous to the conversion from a single to multiple islet autoantibodies in the progression to T1D [41, 42]. In contrast to autoantibodies, CD4 T cell clones isolated from myocarditis heart lesions of DQ8^+^NOD mice predominantly recognized α-MyHC and adoptively transferred disease into immunodeficient hosts, demonstrating that α-MyHC-reactive CD4 T cells cause myocarditis [13••].

Furthermore, using newly developed ex vivo INF-γ ELISPOT assays, autoreactive T cell responses to α-MyHC, but not β-MyHC, were observed in the peripheral blood of healthy humans with markedly increased frequencies of α-MyHC-reactive T cells detected in patients with myocarditis [13••]. These T cell findings were unexpected since α-MyHC only constitutes a small fraction (7–10 %) of the total MyHC in human heart tissue, and challenged longstanding notions based on myocarditis models in mice (in which α-MyHC constitutes >90 % of the total cardiac MyHC) that the immune targeting of α-MyHC was due to its cardiac abundance, and stimulated efforts to define alternative mechanisms.

## Immune Targeting of the Heart Is “Pre-programmed” in the Thymus in Mice and Humans

It is now widely recognized that the thymus, and in particular, specialized stromal cells called medullary thymic epithelial cells (mTECs) play the first and most important role in preventing organ-specific autoimmune disease by their unusual ability to express a wide range of peripheral tissue-specific antigens, leading to the apoptotic elimination (“deletion”) of developing T cells strongly reactive to these self-antigens [43]. This mechanism is perhaps best illustrated by (pro)insulin in T1D, in which decreased thymic expression of insulin is associated with increased susceptibility to T1D in humans (*IDDM2*) [44], and conversely, thymic overexpression of insulin prevents diabetes in NOD mice by deleting insulin-autoreactive T cells [45].

After showing that α-MyHC was unique amongst cardiac antigens in being absent in mTECs, we showed that the transgenic introduction of α-MyHC into the TECs of DQ8^+^NOD mice induced “tolerance” to cardiac myosin (i.e., with the disappearance of T cell and humoral responses to cardiac myosin) and prevented myocarditis [13••]. These results underscored an important role for impaired thymic tolerance mechanisms and demonstrated that α-MyHC is a primary autoantigen in myocarditis. We then showed that healthy humans also normally lack α-MyHC expression in mTECs and that this correlated with augmented T cell responses to α-MyHC in the peripheral blood, particularly in DQ8^+^T1D patients with heart disease. These findings suggested that humanized DQ8^+^NOD mice and T1D patients develop parallel forms of cardiac autoimmunity.

## Role of Inflammation in Myocardial Infarction: a Double Edged-Sword in T1D

One implication of this physiological “hole” in thymic self-tolerance is that healthy individuals should be at risk for developing cardiac autoimmunity after inflammatory heart injury, especially following MI [14] (Fig. 1b). Indeed, transient immune responses to cardiac myosin have been reported in healthy individuals after MI, as was first described over one-half century ago (“Dressler’s syndrome”) [46].

A large body of evidence suggests that acute MI is a highly immunostimulatory event. Following MI, signals are generated that trigger a profound innate inflammatory cascade (Fig. 1b), with influx of dendritic cells, neutrophils, and macrophages and release of proinflammatory cytokines (e.g., TNFα, IL-6, and IL-1) and inflammatory mediators that are key for repair of the infarcted heart [47]. The same necrotic signals and cytokines are also known to be powerful maturative factors for dendritic cells, leading to upregulation of the display of major histocompatibility complex and costimulatory molecules and transforming them into highly immunogenic antigen-presenting cells, capable of initiating antigen-specific immune responses of the adaptive immune system [48].

It has also been suggested that a timely resolution of myocardial inflammation following MI is not a default process, but requires the coordinated activation of multiple inhibitory immune pathways [49–51]. An impairment in the function of these counter-inflammatory responses may affect the healing process and has been implicated in the pathogenesis of ventricular remodeling and heart failure following MI. This “dysfunctional” healing is considered to be responsible for several post-infarction complications including heart failure and poorer outcomes following a MI [50]. We hypothesized that in T1D patients with dysregulated adaptive immune responses, these post-MI autoimmune reactions might become amplified and self-perpetuating, with extension of ischemic injury and pathological consequences [13••, 14].

We will review in the next paragraphs a novel body of evidence supporting such a post-MI autoimmune syndrome in T1D, starting with experimental MI studies in NOD mice and then moving on to studies performed in post-MI patients with T1D.

## Acute MI Induces a Chronic Post-MI Autoimmune Syndrome in NOD Mice

Although the most frequently used MI model of T1D is the streptozotocin-induced diabetes model [52], our group was the first to examine outcomes of MI in a model of autoimmune T1D. Our studies revealed that MI in NOD mice—but not in control B6 mice—induced the appearance of dense lymphocytic myocardial infiltrates beginning in the infarct border zone that normally provides a barrier to contain the post-MI inflammatory response [53]. The infiltrates were notable in having a cellular composition (mainly B220+ B lymphocytes, CD4+ and CD8+ T cells) similar to the spontaneously arising native insulitis lesions of NOD mice [12••]. These findings suggested that similar pathologic processes underlie T1D and post-MI autoimmunity in T1D. Moreover, there was visible and histological evidence of infarct expansion and edema, suggesting that the lymphocytic infiltrates exerted cytotoxic effects and impaired normal scar formation. Over time, the infiltrates extended into areas remote from the infarct zone, with evidence of adverse ventricular remodeling and cardiac enlargement. These findings sharply contrasted with post-MI B6 hearts that showed the expected dense scars and were devoid of infiltrates [12••].

In addition, following MI NOD mice—but not B6 mice—developed high-titer autoantibodies to MyHC and a second cardiac protein, α-actinin 2, a major component of the cardiac z-disc. This post-infarct autoimmune (PIA) syndrome was also characterized by expansion of γ-interferon-producing CD4 T cell responses to α-MyHC (Fig. 1b), similar to the T cell responses observed in DQ8^+^NOD mice with spontaneous myocarditis [12••].

We further demonstrated in humanized DQ8^+^NOD mice that induction of thymic tolerance to α-MyHC eliminated the anti-cardiac immune targeting and thus the development of PIA and enabled normal infarct healing to occur [12••]. This α-MyHC-driven immune response suggested a potential role for antigen-specific tolerance strategies as a possible therapy for T1D patients following MI.

## Development of Novel Immunoassays for Cardiac Autoantibody Detection in Human T1D

The development of PIA in NOD mice and its augmentation in DQ8^+^NOD mice stimulated efforts to determine whether a similar PIA syndrome occurred in T1D patients. However, it soon became apparent that reliable, standardized assays for measuring cardiac autoantibodies were not available, with most laboratories reliant on cadaveric human heart tissues as a source of antigen and some cardiology groups still using indirect immunofluorescence on human heart tissue sections for autoantibody detection, reminiscent of the first islet cell autoantibody assays for T1D four decades ago [54]. In addition, although Western blotting and ELISA techniques were both reliable for MyHC autoantibody detection in NOD mice, we found that human serum performed poorly in these “solid-phase” assay formats with a high false-positivity in serum from healthy control subjects [12••]. Modeling on the success of fluid-phase islet antibody assays that are the gold-standard assay format for diagnosing T1D and for identifying individuals at risk of developing T1D in clinical prevention trials [55], we developed similar radioimmunoprecipitation assays using in vitro transcribed and translated cDNAs encoding full-length (FL) human α-MyHC (*MYH6*), FL β-MyHC (*MYH7*)—the major MyHC isoform expressed in human heart ventricle—human α-actinin 2 (*ACTN2*), and cardiac troponin I (*TNNI3*). With these assays combined, we found that 67 % (12/18) positive control myocarditis patients versus 3/78 (4 %) healthy control sera tested positive, achieving a sensitivity and specificity superior to previously described methods for myocarditis detection [56].

## Cardiac Autoantibody Expression Profiling Differentiates CVD in T1D from T2D

Indeed, using these newly developed assays, we found that T1D patients examined several years following MI (average time of sampling, 4.5 years after a MI) showed persistent titers of cardiac autoantibodies in 15 of 18 (83 %) of cases [12••]. Conversely, the autoantibodies were detectable in only three of 20 (15 %) post-MI T2D patients and three of 78 (4 %) healthy control subjects (T1D post-MI vs T2D post-MI, *P* = 0.0001; T1D post-MI vs healthy controls, *P* < 0.0001) with the post-MI T2D population showing autoantibody responses indistinguishable from healthy controls, *P* = ns).

Of note, we further identified “myocarditis signatures” (i.e., dual autoantibody reactivity to both α- and β-MyHC) between post-MI T1D patients and acute myocarditis patients without T1D or MI that were absent post-MI T2D patients [12••]. Although immune profiling has been extensively used in clinical oncology to subclassify cancers [57], this was the first application of this approach to subclassify CVD in diabetes.

## Novel Use of Cardiac MRI Techniques to Assess Myocardial Inflammation in T1D Patients with Suspected PIA

Over the past two decades, cardiac MRI (CMR) has emerged as the reference standard for measuring left ventricular structure, function, and infarct size [58] that are major predictors of post-MI mortality in the general population [59, 60]. In addition, CMR has emerged as the primary non-invasive technique for assessment of myocardial inflammation in non-diabetic patients with suspected myocarditis [61].

The EDIC study was the first to use CMR to evaluate cardiac function in a large-scale T1D cohort [62•]. Unexpectedly, the prevalence of myocardial scar was only 4.3 % (32 of 741 subjects) and was found in only 19 % of patients with a clinically adjudicated MI. Interestingly, amongst the 713 EDIC participants with no evidence of clinical MI, 21 showed CMR-defined myocardial scars; the great majority of which (14 of 21, 67 %) were of a non-ischemic rather than an ischemic pattern. Although myocardial inflammation was not assessed in this study, non-ischemic scar is a characteristic CMR feature of myocarditis and other inflammatory cardiomyopathies [61].

Our immune profiling studies suggested a chronic myocarditis in a subset of post-MI T1D patients. We therefore tested the feasibility of using cardiac MRI to detect myocardial inflammation in one such patient [12••]. This index case had a history of unexplained decline in cardiac function following a relatively small MI, 6 years prior to enrolling in our study, and was positive for multiple cardiac autoantibodies (including α- and β-MyHC). Cardiac MRI with the conventional contrast agent (gadolinium) showed markedly elevated T2 signal intensity, consistent with myocardial edema from myocarditis. Myocardial inflammation was further demonstrated using the iron-oxide nanoparticle contrast agent, ferumoxytol, which is avidly taken by macrophages. Theses CMR findings raised the possibility that a clinically unrecognized form of chronic myocarditis triggered by MI might contribute to the poor CVD outcomes in T1D [12••]. These translational studies also pointed to new targets for the diagnosis and treatment of T1D heart disease.

## Conclusion

The discovery of post-MI chronic myocarditis syndrome in both humanized animal models and T1D patients opens a new perspective on the etiology, diagnosis, and treatment of CVD in T1D. Our studies provide the first evidence of a T1D disease-specific process, highlighting differences in the pathogenesis of CVD in T1D from that in T2D.

Despite dramatic improvements in CVD outcomes in the general population, excess CVD mortality persists in T1D, even in patients achieving “standard of care”, on-target glycemic control [4••]. Given that MI is the most common cause of heart failure and CVD death, it will be important to perform prospective studies in larger T1D cohorts to determine the impact of cardiac autoimmunity on post-MI outcomes including progression to heart failure. It is surprising how little information is currently available regarding outcomes of T1D patients following MI [7], and only recently has it become more common for papers on CVD in diabetes to distinguish between diabetes types.

Both conventional CMR and newer molecular imaging modalities provide a promising approach to define which cardiac autoantibody profiles are most predictive of myocardial inflammation in T1D. Since chronic myocarditis may be a precursor of heart failure due to dilated cardiomyopathy [63], such information might be used to risk-stratify T1D MI patients and identify which patients would most likely benefit from antigen-specific immunotherapy. It should be noted that clinical trials of MI patients with broad-spectrum immunosuppressive agents to mitigate the post-MI inflammatory response have largely unsuccessful and in fact resulted in worsened post-MI outcomes (with death from MI rupture [64]) underscoring the critical short-term role of the inflammatory response in healing and cardiac repair [50]. Our studies provide the rationale for antigen-specific immune tolerance approaches as a possible therapy for patients with T1D—and potentially for other autoimmune prone individuals in the general population—following MI.

## References

[1] Ryden L, Grant PJ, Anker SD,  Berne C, Cosentino F, Authors/Task Force M (2013). ESC Guidelines on diabetes, pre-diabetes, and cardiovascular diseases developed in collaboration with the EASD: the Task Force on diabetes, pre-diabetes, and cardiovascular diseases of the European Society of Cardiology (ESC) and developed in collaboration with the European Association for the Study of Diabetes (EASD). Eur Heart J.

[2] Harding JL, Shaw JE, Peeters A, Guiver T, Davidson S, Magliano DJ (2014). Mortality trends among people with type 1 and type 2 diabetes in Australia: 1997-2010. Diabetes Care.

[3] Laing SP, Swerdlow AJ, Slater SD, Burden AC, Morris A, Waugh NR (2003). Mortality from heart disease in a cohort of 23,000 patients with insulin-treated diabetes. Diabetologia.

[4.••] Lind M, Svensson AM, Kosiborod M, Gudbjornsdottir S, Pivodic A, Wedel H, et al. Glycemic control and excess mortality in type 1 diabetes. N Engl J Med. 2014;371:1972–82. *This large population-based study was the first to show excess mortality due to CVD, even in T1D patients achieving on-target glycemic control*. 10.1056/NEJMoa1408214 25409370

[5] Nathan DM, Cleary PA, Backlund JY (2005). Intensive diabetes treatment and cardiovascular disease in patients with type 1 diabetes. N Engl J Med.

[6] Nathan DM, Bayless M, Cleary P, Genuth S, Lachin JM, Orchard TJ (2013). Diabetes control and complications trial/epidemiology of diabetes interventions and complications study at 30 years: advances and contributions. Diabetes.

[7] Eckel RH, Eisenbarth GS (2012). Autoimmune diabetes inflames the heart. Sci Transl Med.

[8] Libby P, Nathan DM, Abraham K (2005). Report of the National Heart, Lung, and Blood Institute-National Institute of Diabetes and Digestive and Kidney Diseases Working Group on cardiovascular complications of type 1 diabetes mellitus. Circulation.

[9.••] de Ferranti SD, de Boer IH, Fonseca V, Fox CS, Golden SH, Lavie CJ, et al. Type 1 diabetes mellitus and cardiovascular disease: a scientific statement from the American Heart Association and American Diabetes Association. Circulation. 2014;130:1110–30. *The most comprehensive and up-to-date review on the risk factors for CVD in T1D*. 10.1161/CIR.0000000000000034 25114208

[10] Libby P (2002). Inflammation in atherosclerosis. Nature.

[11] Ross R (1999). Atherosclerosis—an inflammatory disease. N Engl J Med.

[12.••] Gottumukkala RV, Lv H, Cornivelli L, Wagers AJ, Kwong RY, Bronson R (2012). Myocardial infarction triggers chronic cardiac autoimmunity in type 1 diabetes. Sci Transl Med.

[13.••] Lv H, Havari E, Pinto S, Gottumukkala RV,  Cornivelli L, Raddassi K (2011). Impaired thymic tolerance to alpha-myosin directs autoimmunity to the heart in mice and humans. J Clin Invest.

[14] Lv H, Lipes MA (2012). Role of impaired central tolerance to alpha-myosin in inflammatory heart disease. Trends Cardiovasc Med.

[15] Taylor JA, Havari E, McInerney MF, Bronson R, Wucherpfennig KW, Lipes MA (2004). A spontaneous model for autoimmune myocarditis using the human MHC molecule HLA-DQ8. J Immunol.

[16] Deckert T, Poulsen JE, Larsen M (1978). Prognosis of diabetics with diabetes onset before the age of thirty-one. I. Survival, causes of death, and complications. Diabetologia.

[17] Secrest AM, Becker DJ, Kelsey SF, Laporte RE, Orchard TJ (2011). Characterizing sudden death and dead-in-bed syndrome in type 1 diabetes: analysis from two childhood-onset type 1 diabetes registries. Diabet Med.

[18] Secrest AM, Becker DJ, Kelsey SF, Laporte RE, Orchard TJ (2010). Cause-specific mortality trends in a large population-based cohort with long-standing childhood-onset type 1 diabetes. Diabetes.

[19] Soedamah-Muthu SS, Fuller JH, Mulnier HE, Raleigh VS, Lawrenson RA, Colhoun HM (2006). High risk of cardiovascular disease in patients with type 1 diabetes in the U.K.: a cohort study using the general practice research database. Diabetes Care.

[20] Hamman RF (2010). Mortality risk in long-standing type 1 diabetes: hope and concern. Diabetes.

[21] Pambianco G, Costacou T, Ellis D, Becker DJ, Klein R, Orchard TJ (2006). The 30-year natural history of type 1 diabetes complications: the Pittsburgh Epidemiology of Diabetes Complications Study experience. Diabetes.

[22] Marfella R, Esposito K, Giunta R, Coppola G, De Angelis L, Farzati B (2000). Circulating adhesion molecules in humans: role of hyperglycemia and hyperinsulinemia. Circulation.

[23] Livingstone SJ, Looker HC, Hothersall EJ, Wild SH, Lindsay RS, Chalmers J (2012). Risk of cardiovascular disease and total mortality in adults with type 1 diabetes: Scottish registry linkage study. PLoS Med.

[24] Harjutsalo V, Sjoberg L, Tuomilehto J (2008). Time trends in the incidence of type 1 diabetes in Finnish children: a cohort study. Lancet.

[25] Skrivarhaug T, Bangstad HJ, Stene LC, Sandvik L, Hanssen KF, Joner G (2006). Long-term mortality in a nationwide cohort of childhood-onset type 1 diabetic patients in Norway. Diabetologia.

[26.••] Lind M, Bounias I, Olsson M, Gudbjornsdottir S, Svensson AM, Rosengren A (2011). Glycaemic control and incidence of heart failure in 20,985 patients with type 1 diabetes: an observational study. Lancet.

[27] Thygesen K, Alpert JS, Jaffe AS, Simoons ML, Chaitman BR, White HD (2012). Third universal definition of myocardial infarction. Circulation.

[28] American Diabetes Association Clinical Practice Recommendations (2013). Diagnosis and classification of diabetes mellitus. Diabetes Care.

[29] Retnakaran R, Zinman B (2008). Type 1 diabetes, hyperglycaemia, and the heart. Lancet.

[30] Donath MY, Shoelson SE (2011). Type 2 diabetes as an inflammatory disease. Nat Rev Immunol.

[31] Grundy SM, Benjamin IJ, Burke GL, Chait A, Eckel RH, Howard BV (1999). Diabetes and cardiovascular disease: a statement for healthcare professionals from the American Heart Association. Circulation.

[32] Stadinski B, Kappler J, Eisenbarth GS (2010). Molecular targeting of islet autoantigens. Immunity.

[33] Atkinson MA, Eisenbarth GS, Michels AW (2014). Type 1 diabetes. Lancet.

[34] Gale EA (2002). The rise of childhood type 1 diabetes in the 20th century. Diabetes.

[35] Atkinson MA. The pathogenesis and natural history of type 1 diabetes. Cold Spring Harb Perspect Med 2012;2:1-18. 10.1101/cshperspect.a007641 PMC3543105 23125199

[36] Ziegler AG, Nepom GT (2010). Prediction and pathogenesis in type 1 diabetes. Immunity.

[37] Bingley PJ, Christie MR, Bonifacio E, Bonfanti R, Shattock M, Fonte MT (1994). Combined analysis of autoantibodies improves prediction of IDDM in islet cell antibody-positive relatives. Diabetes.

[38] Verge CF, Gianani R, Kawasaki E, Yu L, Pietropaolo M, Jackson RA (1996). Prediction of type I diabetes in first-degree relatives using a combination of insulin, GAD, and ICA512bdc/IA-2 autoantibodies. Diabetes.

[39] Todd JA (2010). Etiology of type 1 diabetes. Immunity.

[40] Elliott JF, Liu J, Yuan ZN, Bautista-Lopez N, Wallbank SL, Suzuki K (2003). Autoimmune cardiomyopathy and heart block develop spontaneously in HLA-DQ8 transgenic IAbeta knockout NOD mice. Proc Natl Acad Sci U S A.

[41] Verge CF, Gianani R, Kawasaki E, Yu L, Pietropaolo M, Chase HP (1996). Number of autoantibodies (against insulin, GAD or ICA512/IA2) rather than particular autoantibody specificities determines risk of type I diabetes. J Autoimmun.

[52] Ziegler AG, Herskowitz RD, Jackson RA, Soeldner JS, Eisenbarth GS (1990). Predicting type I diabetes. Diabetes Care.

[43] Klein L, Kyewski B, Allen PM, Hogquist KA (2014). Positive and negative selection of the T cell repertoire: what thymocytes see (and don’t see). Nat Rev Immunol.

[44] Pugliese A, Zeller M, Fernandez Jr A, Zalcberg LJ, Bartlett RJ, Ricordi C, et al. The insulin gene is transcribed in the human thymus and transcription levels correlated with allelic variation at the INS VNTR-IDDM2 susceptibility locus for type 1 diabetes. Nat Genet. 1997;15:293–7. 10.1038/ng0397-293 9054945

[45] Jaeckel E, Lipes MA, Von Boehmer H (2004). Recessive tolerance to preproinsulin 2 reduces but does not abolish type 1 diabetes. Nat Immunol.

[46] Dressler W (1956). A post-myocardial infarction syndrome; preliminary report of a complication resembling idiopathic, recurrent, benign pericarditis. J Am Med Assoc.

[47] Mann DL, Mann DL (2004). Activation of inflammatory mediators in heart failure. Heart failure, a companion to Braunwald’s Heart Disease.

[48] Matzinger P (1998). An innate sense of danger. Semin Immunol.

[49] Entman ML, Michael L, Rossen RD, Dreyer WJ, Anderson DC, Taylor AA (1991). Inflammation in the course of early myocardial ischemia. FASEB J.

[50] Frangogiannis NG (2014). The inflammatory response in myocardial injury, repair, and remodelling. Nat Rev Cardiol.

[51] Nahrendorf M, Pittet MJ, Swirski FK (2010). Monocytes: protagonists of infarct inflammation and repair after myocardial infarction. Circulation.

[52] Bugger H, Abel ED (2009). Rodent models of diabetic cardiomyopathy. Dis Models Mech.

[53] Frangogiannis NG. The immune system and cardiac repair. Pharmacol Res. 2008;58:88–111. 10.1016/j.phrs.2008.06.007 PMC2642482 18620057

[54] Bottazzo GF, Florin-Christensen A, Doniach D (1974). Islet-cell antibodies in diabetes mellitus with autoimmune polyendocrine deficiencies. Lancet.

[55] Pietropaolo M, Towns R, Eisenbarth GS. Humoral autoimmunity in type 1 diabetes: prediction, significance, and detection of distinct disease subtypes. Cold Spring Harb Perspect Med. 2012;2:1-18. 10.1101/cshperspect.a012831 PMC3475400 23028135

[56] Lappe JM, Pelfrey CM, Tang WH (2008). Recent insights into the role of autoimmunity in idiopathic dilated cardiomyopathy. J Card Fail.

[57] Galon J, Pages F, Marincola FM, Angell HK, Thurin M,  Lugli A (2012). Cancer classification using the Immunoscore: a worldwide task force. J Transl Med.

[58] Hundley WG, Bluemke DA, Finn JP, Flamm SD, Fogel MA, Friedrich MG (2010). ACCF/ACR/AHA/NASCI/SCMR 2010 expert consensus document on cardiovascular magnetic resonance: a report of the American College of Cardiology Foundation Task Force on Expert Consensus Documents. Circulation.

[59] Kwong RY, Sattar H, Wu H, Vorobiof G, Gandla V, Steel K (2008). Incidence and prognostic implication of unrecognized myocardial scar characterized by cardiac magnetic resonance in diabetic patients without clinical evidence of myocardial infarction. Circulation.

[60] Wu E, Ortiz JT, Tejedor P, Lee DC, Bucciarelli-Ducci C, Kansal P (2008). Infarct size by contrast enhanced cardiac magnetic resonance is a stronger predictor of outcomes than left ventricular ejection fraction or end-systolic volume index: prospective cohort study. Heart.

[61] Friedrich MG, Sechtem U, Schulz-Menger J, Holmvang G, Alakija P, Cooper LT (2009). Cardiovascular magnetic resonance in myocarditis: a JACC White Paper. J Am Coll Cardiol.

[62.•] Turkbey EB, Backlund JY, Genuth S, Jain A, Miao C, Cleary PA (2011). Myocardial structure, function, and scar in patients with type 1 diabetes mellitus. Circulation.

[63] Cooper LT (2009). Myocarditis. N Engl J Med.

[64] Roberts R, DeMello V, Sobel BE (1976). Deleterious effects of methylprednisolone in patients with myocardial infarction. Circulation.

[65] Eisenbarth GS (1986). Type I diabetes mellitus. A chronic autoimmune disease. N Engl J Med.

